# Aetiology, Clinical Manifestations, Diagnosis, and Treatment of Oesophageal Perforation: A Review

**DOI:** 10.7759/cureus.55041

**Published:** 2024-02-27

**Authors:** Shaima Shaheem, Hasina Panikkaveettil

**Affiliations:** 1 Internal Medicine, Medical University of Plovdiv, Plovdiv, BGR; 2 Internal Medicine, Our Lady's Hospital, Navan, IRL

**Keywords:** iatrogenic perforation, boerhaave's syndrome, oesophageal stents, oesophageal tear, traumatic esophageal perforation, spontaneous esophageal perforation

## Abstract

Oesophageal perforation (OP) is a life-threatening condition and refers to a tear or disruption in the oesophageal wall. It is considered a medical emergency due to its significant implications, often related to its various causes, such as iatrogenic perforation during endoscopy, Boerhaave syndrome, traumatic injury, foreign body ingestion, and tumour perforation. Early interventions, diagnosis, and a thorough physical examination are essential for better clinical outcomes. Diagnostic procedures and imaging techniques, play a crucial role in confirming OP. The diagnostic workup, based on the index of suspicion, may involve barium oesophagram or contrast-enhanced CT. Once diagnosed, classification of severity using the Pittsburgh clinical severity score guides treatment decisions. Management can be non-surgical or surgical and focuses on a multi-disciplinary approach combining conservative, surgical, or endoscopic methods. Surgical control remains crucial, with the approach dependent on the location of the leak. Improved knowledge of this life-threatening condition is important among healthcare professionals. The objective of this review is to provide information about oesophageal perforation and its early detection, management, and multidisciplinary interventions for optimal patient outcomes.

## Introduction and background

Oesophageal perforation (OP), a potentially fatal condition, may result in unfavourable outcomes when there are delays in diagnosis or inadequate treatment. This condition poses a considerable challenge to the entire interdisciplinary team. The discovery of oesophageal perforation dates back to 1723 when Hermann Boerhaave first described it, noting a spontaneous oesophageal rupture in a Dutch Navy admiral due to recurrent vomiting. Subsequently, in 1947, Barrett and Olson pioneered the initial surgical repair attempts for OP [1]. Oesophageal perforation can manifest in any of the three distinct anatomical compartments, exhibiting varied symptoms, often non-specific, contributing to a significant delay between perforation and definitive diagnosis [2]. Timely intervention within 24 hours is generally considered crucial for improved outcomes, with poorer prognosis associated with perforations related to cancer [3]. Recent studies indicate an overall mortality rate of 20% to 30%, emphasising the strong correlation between the event and intervention [4]. The anatomy of the oesophagus has been described as a muscular tube originating from the foregut, which functions to transport food and liquids from the oropharynx to the stomach. Its passage through the diaphragm occurs at the oesophageal hiatus, positioned at the tenth thoracic vertebral (T10) level [5]. The three different sections of the oesophagus exhibit variations in structure and composition, the organ is approximately 23-25cm in length. The cervical segment ranges from 6-8cm and the thoracic segment extends 15cm, both are characterised by a stratified structure. Abdominal segment ranges 2-3cm and features a columnar structure [6].

## Review

Methodology

**Figure 1:**
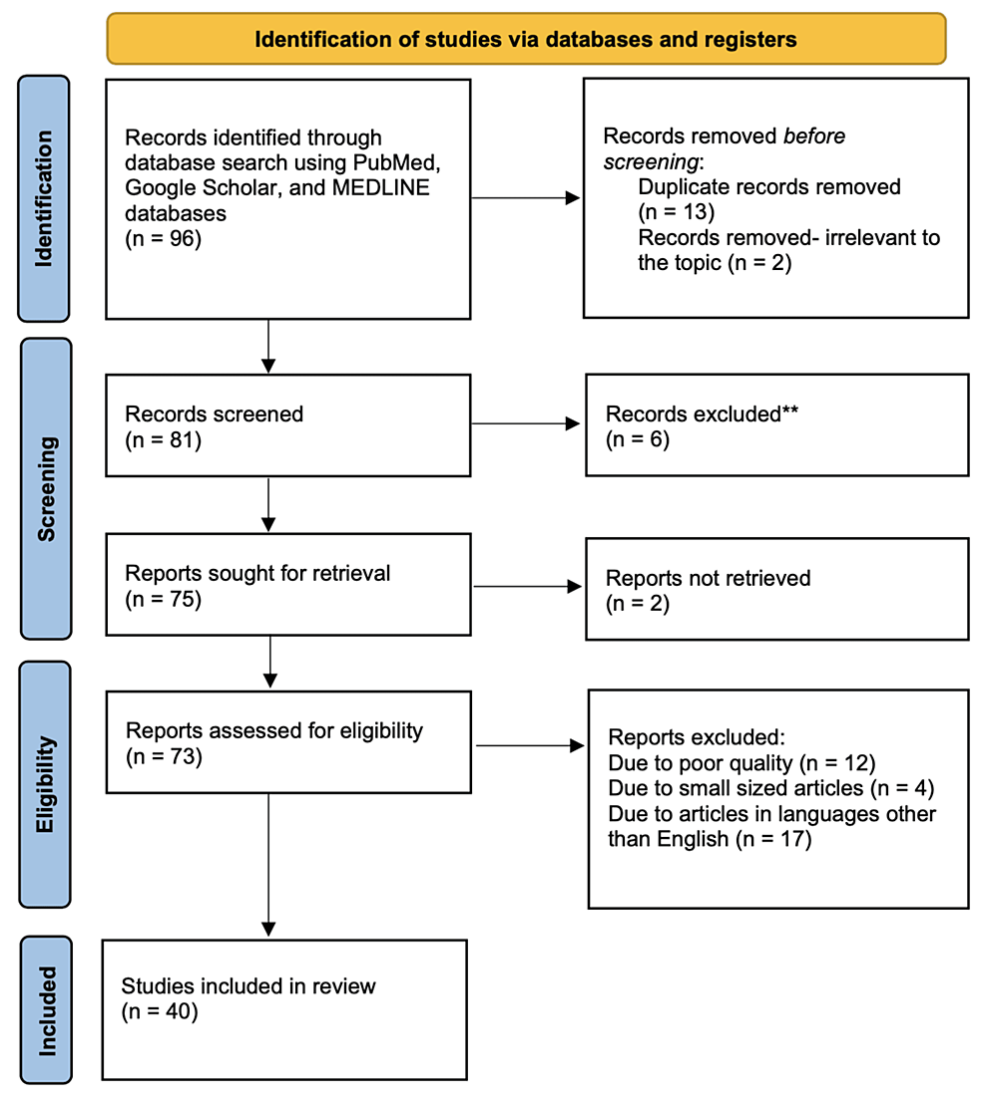
PRISMA methodology by which the study articles were selected. PRISMA: Preferred Reporting Items for Systematic Reviews and Meta-Analyses

In conducting this review, a thorough search was meticulously carried out to identify relevant articles from reputable databases, including PubMed, Google Scholar, and MEDLINE. A comprehensive array of key terms such as "Oesophageal perforation", ‘Aetiology’, ‘Boerhaave syndrome’ and ‘Iatrogenic perforation’ were systematically applied to ensure a comprehensive literature exploration. Only articles in the English language were considered. Exclusions included publications in languages other than English, articles of poor quality, or small-sized publications. Following the initial search, which yielded 96 articles after eliminating duplicates, 81 records were screened and 73 were assessed for eligibility. Ultimately 40 studies were deemed suitable for inclusion in this review. The Preferred Reporting Items for Systematic Reviews and Meta-Analysis (PRISMA) methodology used for searches is depicted in Figure 1.

Aetiology

The primary causes of OP are iatrogenic oesophageal perforations (IOP) and spontaneous perforation, also recognised as Boerhaave’s syndrome (BS). The expanding use of invasive endoscopic oesophageal interventions is associated with the increasing occurrence of IOP [7]. Different causes leading to oesophageal perforation are listed in Table 1.

**Table 1:** Aetiology of oesophageal perforation (OP) Source: [8-10]

Cause	Example
Spontaneous	Forceful retching (Boerhaave syndrome)
Iatrogenic	Endoscopic examinations, Periesophageal surgery
Trauma	Foreign body, Penetrating trauma
Tumour	Oesophageal, Lung, Mediastinal
Infection	Tuberculosis, Acquired immunodeficiency syndrome

Oesophageal perforation, a rare clinical occurrence, stems from various causes, including iatrogenic factors such as endoscopic examinations, surgical procedures, and naso-enteric tube placement. Additionally, non-iatrogenic causes include spontaneous rupture (Boerhaave syndrome), penetrating wounds, foreign body ingestion, and thoracic trauma. Upper gastrointestinal endoscopy stands out as the most prevalent iatrogenic cause leading to perforation [11]. Iatrogenic procedures such as endoscopy, dilatation, and tube insertion have replaced spontaneous rupture as the primary cause of OP, these procedures contribute 60 to 75 percent of oesophageal injuries [12]. Oesophageal perforations can occur in the cervical, thoracic, or abdominal regions. Surgical repair is typically recommended for abdominal perforations, while cervical and thoracic perforations offer the flexibility of either repair or conservative treatment. Cervical perforations tend to have a milder course, but intra-thoracic injuries carry elevated morbidity and mortality, particularly when diagnosed late (after 24 hours). Mortality rates range from 10 to 40 percent, marking a notable improvement from the pre-antibiotic era when rates soared to around 90 percent [13]. The risk of perforation is 0.03 percent for patients undergoing upper endoscopy, which increases to 17 percent with invasive therapies such as radiofrequency ablation or sclerotherapy [14]. Injury frequently occurs during endoscope passage through the cricopharyngeal sphincter, the oesophagus's narrowest zone. Older individuals, often with limited neck extension and osteoarthritic spurs near the posterior oesophageal wall, face heightened risk. The second narrowing site, near the aortic arch and left main bronchus, is more susceptible to perforation from ingested foreign bodies than instruments. The third zone, the gastroesophageal junction, is prone to perforation during biopsy or dilation of benign and malignant strictures or achalasia [15].

Pathogenesis

Oesophageal perforation occurs as an immediate total breach of the oesophageal wall, resulting in the leakage of intraluminal contents into the adjacent mediastinum. This leads to local inflammation, triggering a systemic inflammatory response, and can ultimately progress to sepsis, correlating with elevated morbidity and mortality rates [16]. Sharp objects can cause immediate perforation, whereas blunt foreign bodies may induce pressure necrosis, potentially resulting in delayed perforation [17]. The buccopharyngeal fascia tightly attaches to the posterior walls of the pharynx and oesophagus. Consequently, perforation can breach the retrovisceral space, facilitating the descent of infection into the posterior mediastinum [18]. Perforation in the upper aerodigestive system can lead to serious airway complications as infection can occur in the pre-tracheal space and may descend substernally leading to complications such as pneumothorax, pneumonia, mediastinitis, and retropharyngeal abscess [19]. Upon perforation, immediate diagnosis and treatment must be done. Failure to act promptly may lead to the overflow of digestive juices through the perforation, causing severe inflammation in the peritoneal cavity. This can be life-threatening due to systemic toxic symptoms induced by acute suppurative infection [9].

Clinical manifestations

Symptoms mainly rely on the duration between iatrogenic injury and diagnosis, as well as the location of the perforation. The clinical presentation of oesophageal perforation (OP) is non-specific and may imitate other common conditions such as pancreatitis, pneumonia, and peptic ulcer disease. Common signs include cervical neck, back, or epigastric pain, coupled with difficulties in swallowing (dysphagia), painful swallowing (odynophagia), hoarseness (dysphonia), and laboured breathing (dyspnoea) [20]. The clinical presentation according to location is depicted in Table 2.

**Table 2:** Clinical characteristics of oesophageal perforation (OP) Source: [12,21]

Types	Clinical Presentation
Cervical oesophageal perforation	Cervical pain, neck tenderness, crepitus, dysphagia, odynophagia, dysphonia, subcutaneous emphysema
Thoracic oesophageal perforation	Epigastric or chest pain, crepitus on chest, dyspnoea, cough
Intra-abdominal oesophageal perforation	Abdominal pain, nausea, vomiting

Oesophageal perforation (OP) typically induces sudden and acute pain, often radiating to the back or the left shoulder. The pain is followed by vomiting and dyspnoea in 25% of the patients [22]. OP is associated with the Mackler triad encompassing vomiting, chest pain, and subcutaneous emphysema, however oesophageal perforation seldom manifests with all these symptoms. Patients often present with vague and non-specific complaints, potentially leading to delayed diagnosis and unfavourable outcomes [23]. Upon physical examination, the majority of the patients exhibit significant distress. Tachycardia is frequently observed, with elevated temperature (> 38.5°C) emerging as a later sign. Particular attention should be given to identifying the crepitus as it is indicative of subcutaneous emphysema. A rapid development of systemic inflammatory response typically occurs within 24-48 hours post-perforation, with bacterial mediastinitis posing the risk of development of multiple organ failure, leading to a fatal outcome within a brief period [22]. Untreated perforations may lead to delayed complications, including hypoxia, sepsis, and shock. Thus timely diagnosis and work-up should be carried out to have better clinical outcomes [12].

Diagnosis

Diagnosing and managing oesophageal perforation relies on the early identification of clinical features and accurate interpretation of diagnostic imaging. The prognosis is dependent on variables such as the cause and site of the injury, the existence of other oesophageal conditions, and the duration between perforation and the commencement of therapy. Early diagnosis and intervention are crucial, significantly decreasing mortality by a minimum of 50 percent [24]. Diagnosing within 24 hours from the onset of clinical symptoms was classified as an early diagnosis, while diagnoses made more than 24 hours after symptom onset were deemed late. In a recent study conducted of patients with oesophageal perforation, applying these definitions, early diagnoses were identified in nine cases (60%), all of which resulted in 100 percent survival. In contrast, late diagnosis were observed in six cases (40%), with no instances of survival [25]. While advanced diagnostic techniques have significantly improved modern clinical practice, diagnosing OP remains challenging and can lead to life-threatening complications. Initial diagnosis could be done by direct X-ray and contrast-enhanced tomography, offering crucial insights. Diagnostic endoscopy may also be utilised, predominantly conducted using flexible endoscopes (FE), offering direct visualisation of the oesophagus [26]. Radiologic indications that strongly suggest OP include the presence of air in the soft tissues of the neck, coupled with swelling in the retropharyngeal or retrotracheal areas. Chest X-ray might reveal free air in the mediastinum or cervical space, a widened mediastinum, pneumothorax, or in delayed instances, pulmonary infiltrates. The use of contrast oesophagography is recommended to pinpoint the perforation site and assess the concurrent presence or absence of oesophageal pathology [27]. The most valuable diagnostic test involves a barium swallow study and a gastrografin swallow study [28]. It is crucial to approach OP by considering the severity of the injury (Pittsburgh oesophageal perforation severity score) and the extent of mediastinal and pleural contamination. Non-surgical management can be effectively employed in specific cases, demonstrating minimal morbidity and mortality rates when favourable radiographic and clinical features are evident [29]. The Pittsburgh oesophageal perforation severity score takes into account different clinical factors such as (fever, tachycardia, and leucocytosis, etc.) and radiological factors such as (pleural effusion, and non-contained leak on CT or barium swallow test). A recent study concluded that by utilising the Pittsburgh score, they were able to effectively predict both mortality and morbidity, providing reliable guidance in choosing optimal treatment [30].

Treatment

The choice of treatment is influenced by various factors, such as the cause and site of the perforation, the duration between the triggering event and onset of symptoms, the overall health status of the patient, and the existence of co-morbidities. Due to these considerations, determining the optimal therapeutic approach demands significant physician expertise and good clinical judgment of the physician [31].

Non-Surgical Approaches

Treatment alternatives include both medical and surgical interventions. The criteria recommended for opting for non-surgical treatment are patients who are clinically stable, instrumental perforations identified before significant mediastinal contamination or perforations diagnosed with such a delayed timeframe that the patient has already exhibited tolerance without requiring surgery; and oesophageal disruptions effectively contained within the mediastinum or a pleural loculus [32]. Medical approaches may involve administering antibiotics, intravenous fluids, nasogastric suction, pleural drainage, limited oral intake, and the use of a feeding enterostomy or total parenteral nutrition. As reported in a study, the application of non-surgical therapy in specific cases yielded a 20% mortality rate [33]. Recent evidence suggests that the majority of patients presenting with oesophageal perforation can be effectively treated through non-surgical measures. Individuals with small, clearly defined tears and limited extra-oesophageal involvement may find non-surgical treatment to be a more suitable option. This approach involves establishing a large bore intravenous access, administering oxygen, and implementing cardiopulmonary monitoring within a critical care environment [34]. In contrast to surgical repair, endoscopic closure techniques have evolved as a less invasive approach to management. Options such as through-the-scope clips (TTSCs), over-the-scope clips (OTSCs), and oesophageal stent placement are widely recognised for closure. In cases involving mediastinal collections associated with perforations, a newer option for drainage is endoluminal vacuum therapy (EVT) [35]. Conservative treatment typically leads to favourable outcomes in most cervical perforations. The management of thoracic perforations is closely tied to the perforation size. Conservative treatment is suitable for small perforations, while stent placement may be beneficial for more extensive perforations as long as the disruption of the lumen circumference does not surpass 70 percent [36].

Surgical Approaches

For treatment, surgery continues to be the gold standard, however minimally invasive and endoscopic methods are under exploration. Surgical procedures include primary closure with or without reinforcement with autogenous tissue, simple and T-tube drainage, and oesophageal resection. The principal choice for surgical treatment of thoracic and abdominal oesophageal perforation is primary repair [37]. Although a video-assisted thoracic surgery (VATS) approach has shown success, additional studies are required to understand its role in primary repair [38]. Cervical perforations, typically non-lethal due to containment within surrounding neck structures, can be effectively addressed with a left cervical incision. Often, the management involves drainage of purulent material and potential suturing of the oesophageal mucosa. Injuries to the upper and mid-oesophagus are commonly approached through a right thoracotomy, while distal oesophageal perforations are more efficiently managed via a left thoracotomy [39]. Patients presenting within the initial 24 hours with a non-contained perforation are generally recommended for surgery involving repair, draining of the pleural space and mediastinal debridement. An essential aspect of surgical repair is ensuring adequate soft tissue coverage of the oesophagus. This may involve using a pedicled flap derived from intercostal muscle, pericardial fat, or thickened pleura [40]. In rare instances, for patients with extensive contamination who are unsuitable for primary repair due to tissue friability or pre-existing oesophageal conditions (e.g., inoperable malignancy), diversion procedures or oesophageal resection, including proximal oesophagostomy and feeding gastrostomy or jejunostomy, may be a viable consideration [2]. Following stabilisation, reintroduction of oral feedings is advised, with a contrast oesophagram study verifying oesophageal integrity and the absence of any leakage [20]. A summary of the findings from the various studies included in the review are depicted in Table 3.

**Table 3:** Summary of findings of studies included in this review

Author Name, Year	Findings/Conclusion
Chirica et al., 2010 [1]	Discusses the importance of early diagnosis of oesophageal perforation (OP) and addresses the benefits of non-surgical approaches (medical treatment, endoprostheses and radiological drainage) in selected patients.
Larsen et al., 1983 [3]	Highlighted initiating treatment within 24 hours resulted in decreased mortality rate, emphasising the importance of early surgical intervention.
Liao et al., 2022 [8]	Investigates the safety of non-surgical treatment of OP caused by foreign bodies and concluded its effectiveness even if the perforation is with infection.
Deng et al., 2021 [9]	Examines the recent two-decade trends in the immediate outcomes of OP and draws the conclusion that treatment efficacy has notably improved over the last decade.
Hasimoto et al., 2013 [13]	This study conducted a comparative analysis between conservative and surgical treatments, revealing that surgical intervention demonstrated superior efficacy and safety, particularly in terms of lower mortality rates.
Lampridis et al., 2020 [20]	Emphasised the importance of additional procedures beyond primary repair, such as relief of concomitant obstruction, if there is underlying oesophageal pathology.
Søreide and Viste, 2011 [22]	Proposed considering the patient’s overall condition to choose the most suitable treatment option, underscoring the necessity of a team approach for informed surgical decision-making.
Brinster et al., 2004 [24]	Explores the relation between the rate of morbidity and mortality in oesophageal injuries and factors such as the cause and site of the injury, the presence of underlying pathology, the delay in diagnosis, and the chosen method of treatment.
Shahriarirad et al., 2023 [26]	Discusses different surgical methods of treatment. Given the absence of a gold standard treatment strategy, it is imperative to conduct an individualised assessment for each patient.
Abbas et al., 2009 [29]	Proposed the use of Pittsburgh oesophageal perforation severity score to choose optimal treatment strategies based on clinical and radiological features.
Shaffer et al., 1992 [32]	Provided guidelines for non-surgical treatment selection: 1) Clinically stable patients, 2) Early detection 3) Perforation contained within the mediastinum or a pleural loculus.
Romero and Goh., 2013 [36]	Explores different management methods for different sizes of tears. Small perforations can be managed conservatively while larger perforations can be managed via stent placement.
Nachira et al., 2023 [39]	The study concludes that better outcomes are attained through a multidisciplinary approach, carried out by an expert team, and adopting individualised treatments that encompass all available modalities (medical, endoscopic, and surgical interventions).
Minnich et al., 2011 [40]	Evaluated a prospective algorithm for thoracic oesophageal perforation, revealing that contained perforations can often be safely managed non-surgically with minimal morbidity or mortality. However, active in-hospital monitoring is crucial if surgery is not selected.

## Conclusions

Oesophageal perforation (OP), a life-threatening event, is marked by various causes. The increasing occurrence of iatrogenic oesophageal perforations (IOP) emphasises the need for heightened clinical awareness and precise diagnostic strategies. It is evident that early diagnosis and tailored management are important in averting severe complications, such as multiple organ failure. This underscores the importance of early intervention and emphasises the significance of continuous advancements in diagnostic technologies. Future research should prioritise refining diagnostic approaches to enhance accuracy in diagnosing OP. Investigating the evolving role of minimally invasive and endoscopic methods in the treatment of OP could offer valuable insights for expanding therapeutic options and improving patient outcomes. A multidisciplinary approach, combining medical, endoscopic, and surgical interventions, remains crucial. A comprehensive understanding of the diverse causes and diagnostic and therapeutic modalities is crucial for understanding and managing the risks posed by oesophageal perforation and will ensure the best possible outcomes for affected patients.
